# Perceived work stressors and the transition to burnout among nurses in response to the pandemic: implications for healthcare organizations

**DOI:** 10.5271/sjweh.4148

**Published:** 2024-04-01

**Authors:** Emanuele Maria Giusti, Marco Mario Ferrario, Giovanni Veronesi, Alessia D’Amato, Francesco Gianfagna, Licia Iacoviello

**Affiliations:** 1 EPIMED Research Center, Department of Medicine and Surgery, University of Insubria, Varese, Italy.; 2 School of Medicine, University of Insubria, Varese, Italy.; 3 Mediterranea Cardiocentro, Napoli, Italy.; 4 Department of Epidemiology and Prevention, IRCCS Neuromed, Pozzilli, Italy.; 5 Department of Medicine and Surgery, LUM University, Casamassima, Italy

**Keywords:** health care worker, hospital management, mental health, organizational stressor, prospective longitudinal study

## Abstract

**Objectives:**

This study aimed to assess the associations of pre-pandemic perceived work stressors and work satisfaction among nurses, including nurse assistants, with burnout profiles and their transitions in response to the pandemic.

**Methods:**

Three hundred and thirty-seven nurses working in an Italian University hospital participated in a longitudinal study including a survey in August 2019 investigating perceived work stressors (assessed using the HSE Indicator Tool), work satisfaction (Work Satisfaction Scale), and burnout (Maslach Burnout Inventory), and a second survey in December 2020 assessing burnout. Using latent transition analysis, we identified burnout profiles and then estimated the associations between work stressors and satisfaction on profiles and transitions.

**Results:**

We identified three pre-pandemic profiles, namely *engaged* (67%), *ineffective* (15%), and *burnout* (18%); and three pandemic profiles, namely *engaged* (37%), *exhausted* (51%), and *severe burnout* (12%). The *severe burnout* profile consisted of 70% nurses classified in the *burnout* profile before the pandemic. Overall, work stressors and satisfaction were associated with both pre-pandemic and pandemic *burnout* profiles. Among nurses not in the *burnout* profile prior to COVID-19, pre-pandemic hostile relationships increased [odds ratio (OR) 1.19, 95% confidence interval (CI) 1.05–1.34] and work satisfaction decreased (OR 0.82, 95% CI 0.68–0.98) the probability to transition to *exhausted*. Moreover, work satisfaction (OR 0.54, 95% CI 0.32–0.91) and participation in work organization (OR 0.69, 95% CI 0.51–0.93) protected from transitioning to *severe burnout*. The association between peer support and the transition to *exhausted* needs further investigation.

**Conclusions:**

Pre-pandemic work stressors and satisfaction were associated with pandemic burnout and burnout transitions. To enhance preparedness for future crises, healthcare managers should carefully assess and tackle work-related constraints affecting nurses.

According to the ICD-11, drawing from Maslach’s three-component model (1), burnout is a psychological syndrome characterized by feelings of exhaustion, depersonalization or feelings of cynicism, and a sense of lack of accomplishment (2). Although the debate about the definition of burnout is still ongoing (3, 4), burnout has been recognized as a critical occupational phenomenon, dynamically related but distinct from depression (5–7), with its own distinct pathogenesis (8). It is considered to be an occupational disease in multiple countries (9). Nurses are at risk of developing this syndrome, with estimated pre-pandemic prevalence as high as 11% (10). This is problematic because nurse burnout has been shown to detrimentally affect both their physical and psychological health (11, 12), diminish the quality of care they deliver, jeopardize patient safety (13), and escalate the likelihood of medication errors (14). Notably, burnout prevalence among nurses sharply rose during the COVID-19 pandemic, with half of these workers showing at least one of its symptoms (15). In this context, burnout has been found to reduce their readiness to respond to the crisis and to contribute to high rates of work leave (16, 17), thereby hampering the healthcare system’s ability to react to the pandemic. Hence, understanding burnout determinants among nurses is vital for the dual purpose of safeguarding the well-being of nurses and maintaining preparedness for future crises.

It has been suggested that burnout among healthcare workers may arise from the interplay between the emotional demand intrinsic to their work and the prolonged exposure to work stressors related to organizational dysfunctions (1). Among nurses, perceived work stressors – such as high demands, low control, and hostile relationships – were found to be associated with burnout symptoms, whereas role clarity, managers’ support, and work satisfaction were identified as protective (18). In addition, inadequate staffing, prolonged shift duration, low schedule flexibility, time pressure, and job insecurity have also been reported as contributing to burnout (19). Regarding pandemic burnout, the literature has so far focused on the role of proximal work stressors, including workload, insufficient material and resources, working in COVID-19 designated places, specialized training, and work safety while caring for COVID-19 patients (20, 21). It is worth noticing that most of these factors pertain to the ability to organize work routines and allocate resources, whereas the contribution of pre-existing, chronic work stressors on the transition to burnout has been so far overlooked. In addition, most of this evidence comes from cross-sectional analyses; while longitudinal studies are more suited to separate between pre-existing and pandemic-related factors and then to pursue mitigation actions more carefully.

Several methodological challenges might have impaired the research on this topic so far. First, investigating the association between pre-pandemic work stressors and pandemic burnout requires having collected the relevant data. Second, studying the transition to burnout is challenging due to the multidimensionality of this construct, the above-mentioned debate about its definition (3, 4), and the absence of established clinical thresholds to discriminate people with clinical levels of this syndrome (22, 23). Finally, the role of pre-existing work stressors and work satisfaction should be elucidated under a comprehensive framework that allows simultaneously testing their association with burnout before and during COVID-19, as well as with the probability of developing burnout among nurses initially without burnout. Latent transition analysis (LTA), a person-centered approach that identifies profiles or subgroups of individuals sharing similar response patterns and enables the longitudinal analysis of these profiles and their predictors, can help address these challenges (24).

In the context of a longitudinal study on a representative sample of healthcare workers, we recently found that burnout, defined according to the Maslach model, before COVID-19 and its changes during the pandemic were associated with PTSD symptoms and psychological distress (25). In this paper, using LTA, we aimed to investigate the role of pre-pandemic perceived work stressors and work satisfaction among nurses with burnout profiles and transitions in response to the pandemic.

## Methods

The North Italian Longitudinal Study Assessing the Mental Health Effects of SARS-CoV-2 Pandemic in Health Care Workers is a longitudinal study conducted in a University hospital in the city of Varese, Italy. Between August and September 2019, 1286 doctors, nurses, nurse assistants, and clerks were invited to complete an online survey on work-related stress. Between December 2020 and January 2021, the respondents to the first survey who were still at work (N=717) were invited to a second one assessing the impact of the pandemic. The Institutional Ethics Committee approved the study (approval ID: 69/2020). In the entire sample, participation rates at each wave were 70% and 60%, respectively (26). Among nurses and nurse assistants, who constitute the target population for the current study, participation rates at the first and second waves were 71% (599/844) and 64% (346/541), respectively. Nurses responding to both waves did not differ from those who responded to the first wave only in terms of socio-demographic and working conditions (supplementary material, www.sjweh.fi/article/4148, table S1).

### Assessment of burnout

Burnout was measured in both surveys using a refined version of the Maslach Burnout Inventory (MBI) (27, 28). This questionnaire was selected due to its wide recognition, brevity of administration, global accessibility, validation for use with nurses, and adequate content validity, structural validity, and internal consistency (29, 30). The MBI is a self-report 22-item questionnaire measuring the frequency of attitudes reflecting emotional exhaustion, depersonalization, and poor personal accomplishment on a Likert scale ranging from 0 (never) to 6 (every day). The MBI was refined by Giusti et al (28), who showed that a reduced 18-item version had adequate internal consistency, structural validity, and longitudinal invariance in healthcare workers. Higher subscale scores correspond to higher levels of the corresponding burnout component. Additional details about the refined MBI, including a description of its subscales, their range, and a sample item of each of them are reported in supplementary table S2. In this study, the Cronbach’s α of the emotional exhaustion, depersonalization, and poor personal accomplishment subscales were 0.88, 0.71, and 0.72, respectively.

### Assessment of perceived work stressors and work satisfaction

The predictor variables were perceived work stressors and work satisfaction measured in the 2019 survey. Work stressors were assessed using the Health and Safety Executive Management Standards Indicator Tool (HSE) (31), a validated self-report measurement instrument evaluating work conditions linked to work stress and recommended measure of perceived work stressors by the Italian Workers’ Compensation Authority (INAIL) (32). The HSE includes 35 items on a 1 (never) to 5 (always) Likert scale. We employed the seven subscales (demands, control, peer support, managers’ support, role clarity, participation in work organization, hostile relationships) identified in a previous study on the factor structure of the HSE among healthcare workers (26). Since the HSE follows a formative measurement model, and consequently the assumptions for the calculation of internal consistency indices are not met, the Cronbach’s α of the components was not calculated (26, 33). Details about the number of items of each subscale, as well as their description, their range and sample items are reported in supplementary table S2.

Work satisfaction was measured using a Work Satisfaction Scale including four items assessing satisfaction level on a 1 (unsatisfied) to 4 (very satisfied) Likert scale. This scale was purposefully developed for this study by adapting two items from the Copenhagen Psychosocial Questionnaire (34) referring to work prospects and how personal abilities are used, and adding two items referring to work results and salary (26). Higher scores indicate higher levels of work satisfaction. The Work Satisfaction Scale showed adequate internal consistency and structural validity (26). Additional details about the scale are reported in supplementary table S2. In this study, the Cronbach’s α of the scale was 0.73.

### Statistical analyses

From the original sample of 346 nurses participating in both study waves, we excluded N=9 (2.6%) due to missing data, leaving a final sample size of 337. The socio-demographic and work variables were described using counts and percentages. Then, data were analyzed using LTA. Briefly, LTA is a statistical method that enables the identification of profiles (ie, subgroups of individuals with similar patterns of responses) in multiple waves of longitudinal studies, to assess the invariance of those profiles over time as well as the association between covariates and the probability of being in each profile and transitioning to a different profile over time. In this study, we employed the scores of the three MBI subscales to identify the burnout profiles.

First, MBI data were analyzed separately at each time point to decide the number of profiles to extract. Starting from two profiles, one profile at a time was added and retained if all the following criteria were satisfied: (i) its addition improved the model fit according to a bootstrapped likelihood ratio test, (ii) in the resulting model no profile included <25 nurses, (iii) the entropy of the resulting model was >0.80, which is a standard threshold indicating that the identified profiles are clearly separated from the perspective of the posterior probabilities of classification, and (iv) based on content-related considerations, ie, whether the identified profiles hold theoretical value and can be conceptually distinguished from one another (35). Profiles were interpreted according to the mean scores of each dimension, as previously reported in the literature (36).

Then, the longitudinal measurement invariance of the retrieved profiles was assessed by comparing the fit of a model in which the parameters of the profiles were constrained to be equal over time with the fit of an unconstrained model, using a scaled chi-square difference test based on log-likelihood values (24). The model resulting from this analysis was employed to assess the frequencies of the transitions between profiles over time.

The role of work stressors and work satisfaction scores on burnout profiles was assessed using a comprehensive approach in which covariates were added in nested models at increasing complexity. Covariates were incorporated using Vermunt’s three-step approach (37), described in the supplementary material. At each step, the more complex model was retained based on improvement in the log-likelihood, determined using a scaled chi-square test, as compared to the previous one. A depiction of the analyzed LTA models is reported in supplementary figure S1. First, we developed a reference transition model including only the profiles identified before and during COVID-19. In model 1, the work stressors and the work satisfaction scores were added to the reference model as predictors of profile membership before COVID-19. In model 2, these scores were employed as predictors also of profile membership during COVID-19. Then, we evaluated the role of additional covariates (age, sex at birth, and job title) by adding them to the retained model based on the previous steps. Finally, among those who were not in the burnout profile at baseline, we performed a multinomial logistic regression to assess the role of work stressors and work satisfaction in influencing profile transitions. The analyses were performed using SAS, version 9.4 (SAS Institute Inc, Cary, NC, USA) and Mplus, version 7 for LTA modeling.

## Results

The flow of participants has been documented in previous articles (26, 28). The socio-demographic and work-related characteristics of the sample are reported in table 1. Most of the sample was composed of nurses (N=256, 76.0%), females (N=279, 82.8%), aged 45–54 years (N=156, 46.3%), and a work seniority of more than 16 years of work (N=151, 44.1%). More than half of the sample had worked in a COVID-19 ward at the time of the second survey (N=190, 56.4%).

**Table 1:** Demographic and work-related characteristics of the sample (N=337) Variable

		N	%
Sex at birth			
	Female	279	82.79
	Male	58	17.21
Age groups (years)			
	25–34	58	17.21
	35–44	71	21.07
	45–54	156	46.29
	>55	52	15.43
Educational attainment			
	Primary school	43	12.76
	Secondary school	147	43.62
	University degree	120	35.61
	Postgraduate programs	27	8.01
Job title			
	Nurse	256	75.96
	Nurse assistant	81	24.04
Work seniority (years)			
	<5	94	27.89
	5–6	92	27.30
	>16	151	44.1
Worked in a COVID-19 ward			
	No	147	43.62
	Yes	190	56.38

### Identification of the burnout latent profiles

**Figure 1:**
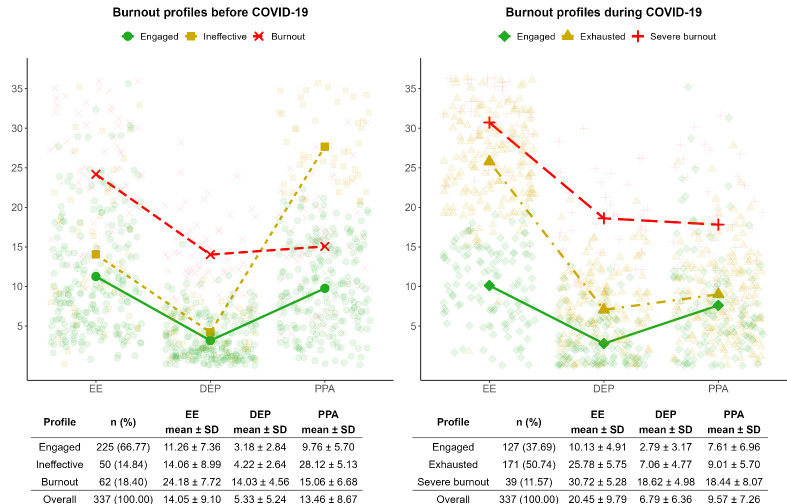
Representation of the burnout profiles before (left panel) and during (right panel) the COVID-19 pandemic, identified through latent transition analysis. [EE=emotional exhaustion; DEP=depersonalization; PPA=poor personal accomplishment.]

We identified three profiles before COVID-19 and three profiles during COVID-19. The longitudinal measurement invariance did not hold, therefore the mean values of emotional exhaustion, depersonalization, and poor personal accomplishment in the profiles before and during COVID-19 were allowed to vary over time. Additional details regarding these analyses are reported in supplementary tables S3 and S4. Figure 1 reports the prevalence and mean values of each burnout profile. Profiles before COVID-19 were *engaged* (66.8%), which includes nurses characterized by low levels of emotional exhaustion, depersonalization, and poor personal accomplishment; *ineffective* (14.8%), showing low levels of emotional exhaustion and depersonalization, and high levels of poor personal accomplishment; and *burnout* (18.4%), comprising nurses with the highest mean levels of emotional exhaustion and depersonalization, and poor personal accomplishment levels positioned between those observed in the *engaged* and *ineffective* profiles. During COVID-19, we identified an *engaged* profile (37.7%) similar to the one identified before COVID-19; a profile characterized by the highest mean levels of emotional exhaustion only, labeled as *exhausted* (50.7%), and a third profile characterized by the highest mean levels of emotional exhaustion, depersonalization, and poor personal accomplishment, labeled as *severe burnout* (11.8%) since these mean levels were higher than the corresponding values of the pre-pandemic *burnout* profile.

Among *engaged* nurses before COVID-19, 47.6% remained in the *engaged* profile during COVID-19, 50.1% transitioned to *exhausted*, and 2.3% transitioned to *severe burnout*. Among *ineffective* nurses before COVID-19, 34.5% transitioned to *exhausted* and 20.6% to *severe burnout*. Finally, 44.8% of the nurses in the *burnout* profile before COVID-19 transitioned to *exhausted*, and 49.8% to *severe burnout*. Therefore, the *severe burnout* profile consisted of 70.0% subjects previously in the *burnout* profile, 25.0% in the *ineffective* profile, and 5.0% in the *engaged* profile.

### Association between work stressors and work satisfaction with burnout profiles

The results of the analysis performed to assess the role of work stressors and work satisfaction on burnout profiles are reported in table 2. Adding the work stressors and work satisfaction as predictors of profile membership before COVID-19 (model 1) improved the model fit. Adding to model 1 a direct relationship between these covariates and the profile membership during COVID-19 (model 2) further improved the model fit. The addition of sex at birth, age, and job title did not improve further the model fit (log-likelihood=521.82, scaled chi-square test value = 27.34, P=0.13). Therefore, model 2 was retained, according to which work stressors and work satisfaction have a direct effect on MBI profiles both before and during COVID-19.

**Table 2:** Changes in model fit following the addition of covariates to the latent transition analysis model.

Model number	Description	Log likelihood	Model fit improvement test (from reference model)	
			Chi-square	P-value
Reference	Three profiles before and during COVID-19, no covariates	-598.23		
1	Direct effects of work stressors and work satisfaction on profiles before COVID-19	-561.35	64.80	<0.001
2	Direct effects of work stressors and work satisfaction on profiles before and during COVID-19	-537.39	45.66	<0.001

Table 3 reports the odds ratios (OR) with 95% confidence intervals (CI) representing the relationships between the work stressors and work satisfaction scores and the profiles before and during COVID-19. Before COVID-19 (column 3), demands were associated with a higher likelihood of being in the *burnout* profile (OR 1.11, 95% CI 1.01–1.21), whereas role clarity (OR 0.76, 95% CI 0.63–0.93) and work satisfaction (OR 0.70, 95% CI 0.54–0.92) with a lower likelihood of being in that profile. Conversely, peer support (OR 1.21, 95% CI 1.03–1.43) and hostile relationships (OR 1.19, 95% CI 1.01–1.41) were associated with a higher probability and work satisfaction (OR 0.80, 95% CI 0.67–0.99) with a lower probability of being *exhausted* rather than *engaged* during COVID-19 (column 6).

**Table 3:** Odds ratios (OR) for the associations between perceived work stressors and work satisfaction with the burnout profiles before and during COVID-19. [CI=confidence interval].

	Before COVID-19				During COVID-19		
	Profile	OR ^a^	95% CI		Profile	OR ^a^	95% CI
Demands	Engaged	Ref			Engaged	Ref	
	Ineffective	0.88	0.78–1.01		Exhausted	1.04	0.95–1.15
	Burnout	1.11	1.01–1.21 ^b^		Severe burnout	0.98	0.82–1.16
Control	Engaged	Ref			Engaged	Ref	
	Ineffective	0.93	0.80–1.09		Exhausted	0.95	0.84–1.07
	Burnout	1.06	0.92–1.22		Severe burnout	1.11	0.93–1.33
Role clarity	Engaged	Ref			Engaged	Ref	
	Ineffective	0.88	0.71–1.08		Exhausted	0.98	0.79–1.22
	Burnout	0.76	0.63–0.93 ^b^		Severe burnout	0.76	0.51–1.14
Managers’ support	Engaged	Ref			Engaged	Ref	
	Ineffective	1.00	0.76–1.30		Exhausted	1.12	0.93–1.34
	Burnout	1.10	0.94–1.28		Severe burnout	1.01	0.77–1.32
Peer support	Engaged	Ref			Engaged	Ref	
	Ineffective	0.93	0.75–1.17		Exhausted	1.21	1.03–1.43*
	Burnout	1.05	0.86–1.29		Severe burnout	1.16	0.90–1.48
Hostile relationships	Engaged	Ref			Engaged	Ref	
	Ineffective	0.95	0.81–1.13		Exhausted	1.19	1.01–1.41 ^b^
	Burnout	1.19	0.97–1.46		Severe burnout	1.13	0.81–1.57
Participation in work organization	Engaged	Ref			Engaged	Ref	
	Ineffective	0.95	0.82–1.10		Exhausted	0.90	0.79–1.04
	Burnout	1.04	0.91–1.20		Severe burnout	0.82	0.67–1.02
Work satisfaction	Engaged	Ref			Engaged	Ref	
	Ineffective	0.78	0.58–1.04		Exhausted	0.80	0.67–0.99 ^b^
	Burnout	0.70	0.54–0.92 ^b^		Severe burnout	0.78	0.49–1.22

^a^ The OR were estimated within the framework of a latent transition analysis model that specifies direct effects of work stressors and work satisfaction on profiles before and during COVID-19 (model 2 in the main text). ^b^ P<0.05

### Role of work stressors and work satisfaction on transition probabilities

Table 4 reports the OR of transition to *exhausted* or to *severe burnout* profiles during COVID-19, amongst nurses who were *engaged* or *ineffective* before COVID-19 (N=275). Pre-pandemic hostile relationships (OR 1.19, 95% CI 1.05–1.34) were positively associated and work satisfaction (OR 0.82, 95% CI 0.68–0.98) was negatively associated with transitioning to ’*exhausted*. In addition, work satisfaction (OR 0.54, 95% CI 0.32–0.91) and participation in work organization (OR 0.69, 95% CI 0.51–0.93) protected from transitioning to ’*severe burnout*. Finally, peer support favored the transition to *exhausted* (OR1.17, 95% CI 1.02–1.34), in particular among *engaged* nurses before COVID-19 (OR 1.20, 95% CI 1.04–1.39; supplementary table S5).

**Table 4:** Odds ratios (OR) of transitioning to *exhausted* or *severe burnout* profiles during COVID-19, amongst nurses who were *engaged* or *ineffective* before the pandemic (N=275). [CI=confidence interval]

	Transition to *exhausted* during COVID-19			Transition to *severe burnout* during COVID-19	
	OR ^a^	95% CI		OR ^a^	95% CI
Demands	1.04	0.97–1.11		0.81	0.65–1.01
Control	0.97	0.89–1.05		1.13	0.89–1.44
Role clarity	1.01	0.89–1.15		0.73	0.51–1.05
Managers’ support	1.08	0.97–1.22		1.05	0.80–1.38
Peer support	1.17	1.02–1.34 ^b^		1.10	0.77–1.57
Hostile relationships	1.19	1.05–1.34 ^b^		1.24	0.85–1.81
Participation in work organization	0.93	0.84–1.03		0.69	0.51–0.93 ^b^
Work satisfaction	0.82	0.68–0.98 ^b^		0.54	0.32–0.91 ^b^

^a^ OR were calculated using a multinomial logistic model, with the Engaged profile during the COVID-19 pandemic as the reference category. ^b^ P<0.05.

## Discussion

Most studies on burnout during the pandemic focused on the ability of healthcare systems to react to the acute phases of the emergency, mostly in terms of work routines and resource allocation (38). These aspects are important but provide limited information on how to build a proactive organization capable of effectively handling future crises. To this extent, the novelty of our paper is three-fold. First, we showed that burnout profiles during the pandemic are linked to the pre-pandemic ones. Nurses who were already *ineffective* or in *burnout* before the pandemic had the highest probability to experience *severe burnout* during the pandemic, while this probability was only 2% amongst the *engaged*. Hence, only a fraction of the high prevalence of burnout during the COVID-19 pandemic reported in the literature (15) are new cases. Second, we showed that pre-existing work organization-related perceived stressful and protective conditions influenced burnout profiles in response to an acute crisis such as the pandemic. Finally, we found that perceived work stressors and work satisfaction were associated with the probability of transitioning to *exhausted* or *severe burnout* during the pandemic in those who were burnout-free before COVID-19. Altogether, these results imply that pre-existing work conditions hindered individuals’ ability to respond effectively to the healthcare crisis and influenced the risk of developing burnout.

To the best of our knowledge, this study is the first to apply LTA to assess the predictors of burnout in response to a healthcare crisis like the COVID-19 pandemic. The profiles identified as *engaged*, *ineffective*, *exhausted*, and *burnout* have consistently appeared, albeit with minor variations, across previous studies employing latent profile analyses or LTA to assess burnout in healthcare workers or other populations (36, 39). In contrast to these studies that were conducted before the pandemic, we identified a *severe burnout* profile, which clustered those who suffered the most from the pandemic. In addition, we found that the longitudinal measurement invariance of the profiles was not met, indicating that between the first and the second wave distinct profiles emerged. The LTA aims to identify different subpopulations within an overarching population. In this context, the lack of measurement invariance indicates that the composition or characteristics defining these subpopulations altered significantly across the observed waves, signifying potential shifts or changes within the underlying burnout subgroups over time (36). In particular, the absence of the *ineffective* profile during COVID-19 aligns with the findings of a previous study in the same cohort of nurses that reported improvements in poor personal accomplishment, potentially attributable to the increased social recognition experienced during the pandemic (25). Conversely, the identification of the *exhausted* profile, which was the one with the highest frequency during the pandemic and mainly included nurses who were previously *engaged*, might reflect the effect of the emotional burden attributable to the healthcare crisis.

Almost all the OR for work satisfaction, role clarity, and participation in work organization were less than one, and some were statistically significant. This suggests that these variables might protect from being in, or transitioning to, a detrimental burnout profile. These factors were found to be protective in studies performed before the pandemic, due to their effect in reducing the perceived workload and increasing motivation, engagement, personal well-being, and enhancing team effectiveness (40–42). Work satisfaction seems to play a pivotal role, as it demonstrated consistent associations with profile memberships and transitions. Role clarity was associated only with burnout profile membership before COVID-19. Participation in the work organization was not associated with profile membership before and during COVID-19 but protected from transitioning to *severe burnout*. Enhancing role clarity and organizational participation, along with the associated work satisfaction, should ideally be nurtured within the workforce before crises occur.

Hostile relationships before COVID-19 had a negative impact on burnout profile membership or transitions (most OR higher than one). The detrimental effect of conflicting relationships on burnout is well established in the literature, and therefore its detection and contrast should be considered a target to improve well-being in work organizations (19). In contrast to other studies, we did not observe a protective relationship of pre-pandemic peer support on pandemic burnout (43). Throughout the investigated pandemic period (2020), employees were assigned almost daily to different hospital wards to meet urgent organizational needs. These frequent changes, coupled with personnel shortages and the emergency setting, might have unsettled relationships between colleagues. Nurses who benefitted more from peer support before the pandemic might have been more likely to experience burnout during the pandemic. Since we did not measure peer support during the pandemic, caution should be used before concluding that it played a negative role on burnout based on our results. Interestingly, in our study, participation in work organization and work satisfaction reflect nurses’ characteristics whose protective effects on burnout are less prone to change, even in periods of exceptional constraints in working conditions. Future studies examining peer support as well as other psychosocial work stressors among workers experiencing frequent team modifications, using repeated measurements, may be better suited to further explore the relationships that we observed here.

The main strength of this study lies in its longitudinal design, which enabled the investigation of the impact of pre-existing work stressors on burnout development. Furthermore, the use of LTA overcame the absence of validated thresholds for the MBI and mitigated the potential overestimation of mental health disorders during the pandemic that can occur when employing pre-specified cut-offs (28, 44). The main limitation concerns the generalizability of the findings to different periods since they refer to a specific time frame of the pandemic, namely the second COVID-19 wave, before the vaccination period. In addition, work stressors and work satisfaction were not measured during COVID-19, limiting the possibility to assess whether their changes influenced burnout during the pandemic. This choice was adopted to avoid an excessive length of the questionnaire administered during the pandemic that might have reduced participation. Nonetheless, it is important to highlight that a longitudinal study that encompassed multiple measurements both before and after the pandemic did not find substantial changes in perceived work stressors (21). Finally, the sample size coupled with the low probability of some transitions prevented the assessment of the role of work stressors on all the transitions in a single, comprehensive LTA model. Consistent with the aim of our study, we partially overcame this limitation by examining the impact of work stressors on transitions among *engaged* and *ineffective* nurses.

### Concluding remarks

In conclusion, the large majority of severe burnout during the pandemic among nurses is determined by pre-pandemic conditions of lack of personal accomplishment or overt burnout. Perceived work stressors and work satisfaction play a key role on burnout profiles before and during the pandemic, as well as on the transition to *exhausted* or to *severe burnout* during the pandemic among previously burnout-free nurses. In the healthcare setting, future crises’ mitigation should begin before the crises outbreak.

To this end, the implementation of interventions that tackle structural work organizational constraints, like insufficient staffing and a poor work environment as well as changes in working time arrangements, seem to be preferred by healthcare workers rather than interventions aimed at improving their psychological response to work constraints (16) and showed to be effective for the prevention of burnout (45). Our results underscore the importance of some selected targets for interventions: enhance participation in work organization through engaging nurses in the decision-making process; increase work satisfaction through providing clear career perspectives based on acquired merits; and improve relationships within the workplace through monitoring harassment, bullying, and violences (46). We provide sufficient evidence to implement these as long-term practices or operating standard procedures in healthcare organizations.

## Supplementary material

Supplementary material

## Protection of research participants

This study was approved by the Research Ethics Committee of the ASST Sette Laghi (approval ID: 69/2020). Prior to participation, all individuals provided their informed consent by signing a consent form. The study was conducted in accordance with the principles outlined in the Helsinki Statement.
