# Supplementary material for: Perceived work stressors and the transition to burnout among nurses in response to the pandemic: implications for healthcare organizations
Source: Scand J Work Environ Health. 2024 Mar 27;50(3):158–67. doi: 10.5271/sjweh.4148 (PMC11014740; doi:10.5271/sjweh.4148)
Supplement: Supplementary material [file SJWEH-50-158-S001.pdf]

Perceived work stressors and the transition to burnout among nurses in response to the pandemic: implications for healthcare organizations[1]

by Emanuele Maria Giusti, PhD, Marco Mario Ferrario, PhD,[2] Giovanni Veronesi, PhD, Alessia D'Amato, PhD, Francesco Gianfagna, PhD, Licia Iacoviello, PhD

1. *Supplementary material*
2. *Correspondence to: Dr Marco Mario Ferrario, EPIMED Research Center, Department of Medicine and Surgery, University of Insubria, 21100 Varese, Italy. [E-mail: marco.ferrario@uninsubria.it]*

Socio-demographic and work-related characteristics of respondents at both study waves and respondents at the first wave only.

Table S1. Socio-demographic and work-related characteristics assessed at the pre-COVID-19 wave, for nurses and nurse assistants who responded and who did not respond at the COVID-19 wave.

	Respondents (N=346)	Not respondents (N=195)	p-value^
**Age, years**	45.4±9.5	44.7±10.1	0.48
**Men, n (%)**	59 (17.1%)	42 (21.5%)	0.20
**Educational attainment, n (%)**			
Less than high school	44 (12.7%)	33 (16.9%)	
High school	151 (43.6%)	71 (36.4%)	0.18
University degree	151 (43.6%)	91 (46.7%)	
**Work seniority, years**	15.6±10.7	14.2±11.1	0.13
**Type of employment, n (%)**			
Full-time	291 (84.1%)	174 (89.2%)	0.10
Part-time	55 (15.9%)	21 (10.8%)	
**Work schedule, n (%)**			
Day-time work	30 (8.7%)	13 (6.7%)	
Shift work w/o night shift	71 (20.5%)	42 (21.5%)	0.70
Shift work with night shift	245 (70.8%)	140 (71.8%)	
**Hospital ward, n (%)**			
Emergency Department	158 (45.7%)	95 (49.0%)	
Medical wards	63 (18.2%)	44 (22.7%)	0.15
Surgical wards	125 (36.1%)	56 (28.7%)	

Note. The table reports mean±standard deviation for continuous variables, and n (%) for categorical variables.
^: p-value comparing respondents to not respondents, from t-test for continuous variables and chi-square test for categorical variables.

Details about the measurement instruments

Table S2 reports the subscales of each of the measurement instruments employed in this study, along with their description, the number of items, their range, and a sample item.

Table S2. Description of the measurement instruments employed in the current study

Instrument	Subscale	Description	Number of items	Range	Sample item
Maslach Burnout Inventory - revised[a]	Emotional exhaustion	Feelings of being emotionally overextended and depleted of emotional resources	6	0-36	"I feel emotionally drained from my work"
	Depersonalization	Impersonal response toward recipients of the worker's care or service	5	0-30	"I feel like I treat some recipients as if they were impersonal objects"
	Poor personal accomplishment	Feelings of incompetence and a decline in one's accomplishments at work	7	0-42	"I deal very effectively with the problems of my recipients"[b]
Health and Safety Executive Management Standards Indicator Tool[c]	Demands	Issues associated with workload and work patterns	8	8-40	"I have unachievable deadlines"
	Control	Autonomy in organizing one's own work	5	5-25	"I have some say over the way I work"
	Peer support	Accessibility of colleagues to provide assistance in work-related issues	4	4-20	"If work gets difficult, my colleagues will help me"
	Managers' support	Encouragement and perceived availability of managers to provide help if needed	4	4-20	"I am given supportive feedback on the work I do"
	Role clarity	Degree of understanding of the worker's role within the organization	5	5-25	"I am clear what is expected of me at work"
	Participation in work organization	Worker's involvement in decisions about their own work or about organizational changes	5	5-25	"Staff are always consulted about change at work"
	Hostile relationships	Being involved in conflicts with, or being	4	4-20	"Relationships at work are strained"

	subject to bullying or harassment by, peers or managers in the workplace			
Work Satisfaction Scale[d]	Satisfaction regarding work prospects, how personal abilities are used, work results and salary	4	4-16	“How pleased are you with your work prospects?”

Note. [a] Scale revised as described in Giusti et al., 2022 (28). [b] The responses to this subscale were reversed. [c] The subscales were identified using a Principal Component Analysis described in Veronesi et al., 2022 (26). [d] The scale is described in Veronesi et al., 2022 (26).

Details and representation of the analyses performed to assess the role of work-related stressors and work satisfaction

The incorporation of the covariates in the analysis of the LTA models assessing the role of work-related stressors on burnout profiles was performed using Vermunt’s three-step approach. This approach was chosen to ensure that the addition of covariates did not influence the characteristics and composition of the burnout profiles. The three steps were performed as follows. First, based on the previous analyses, two latent profile models extracting three profiles were fitted separately using the MBI responses before and during COVID-19. Second, subjects were assigned to a latent profile before COVID-19 and to a latent profile during COVID-19 based on their posterior profile membership probabilities. Third, a multinomial logistic regression model was estimated using the profile assignments as the observed dependent variable.

Figure S1 represents the tested LTA models. The models are numbered according to how they are presented in the Results section of the main text and in Table 2. The reference model is the result of separate Latent Profile Analyses performed to assess the number of profiles to extract before and during COVID-19 and an analysis performed to assess if the profiles are invariant over time. Model 1 posits a direct effect of work-related stressors and work satisfaction on profile membership before COVID-19. Model 2 also posits a direct effect of work-related stressors and work satisfaction on profile membership during COVID-19.

Figure S1. Representation of the Latent Transition Analysis models employed to assess the role of work-related stressors and work satisfaction

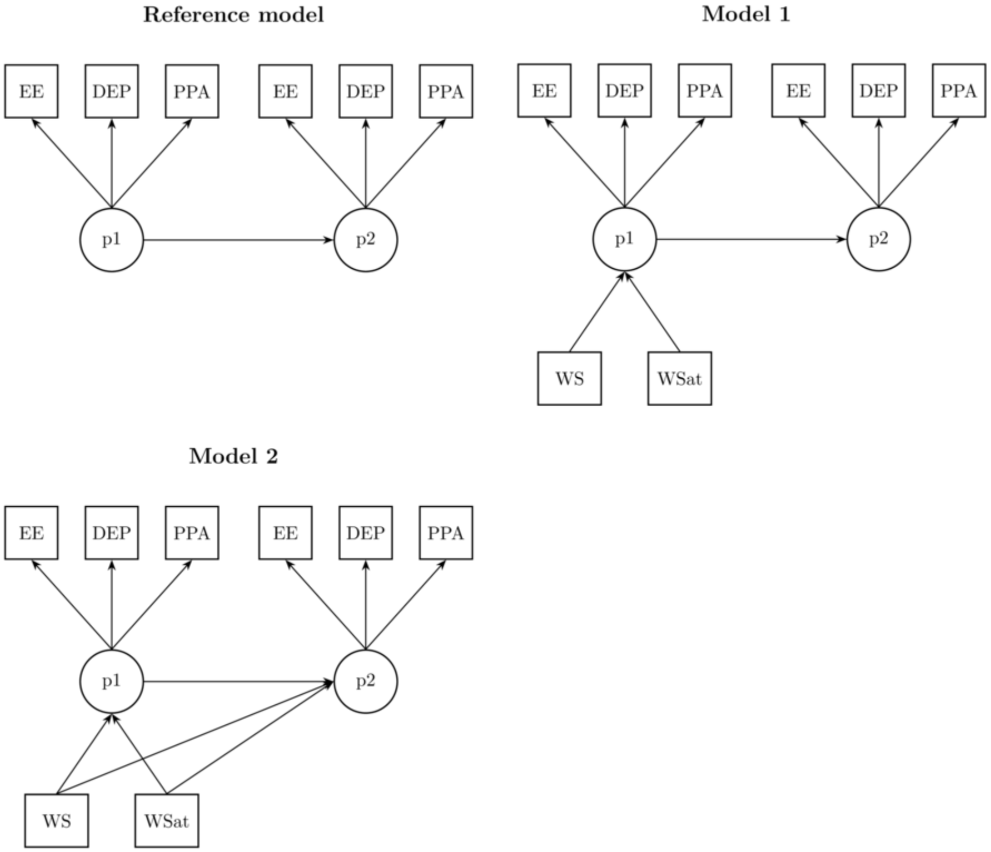

Note. Abbreviations: p1=profiles before COVID-19, p2=profiles during COVID-19, EE=Emotional Exhaustion, DEP=Depersonalisation, PPA=Poor Personal Accomplishment, WS=Work Stressors, WSat=Work Satisfaction.

Supplementary results

Identification of the latent profiles and measurement invariance

The results of the latent profile analysis performed to identify the burnout profiles before COVID-19 showed that extracting three and four profiles resulted in a better fit compared to the respective previous model (Table S3). In the model with four profiles, however, one of the profiles included only 22 subjects. Three profiles were therefore extracted from the burnout scores before COVID-19.

Table S3. Comparisons between the models identified using the Latent Profile Analysis performed to assess the number of burnout profiles before COVID-19 to be extracted

					Bootstrapped likelihood ratio test		
Model	Profile	n	%	Entropy	Log-likelihood	Log-likelihood difference*2	p-value
2 profiles	1	240	63.6	.69	-2395.66	-	-
	2	137	36.4				
3 profiles	1	247	65.5	.81	-3418.36	46.26	<0.001
	2	61	16.1				
	3	69	18.4				
4 profiles	1	230	61.1	.84	-3386.30	47.07	<.0.001
	2	57	15.2				
	3	75	19.9				
	4	22	5.90				

Similarly, the results of the latent profile analysis performed to identify the burnout profiles during COVID-19 showed that extracting three or four profiles improved the fit compared to the respective previous model, but extracting four profiles resulted in a model with a profile with relative frequency <25 (Table S4). Therefore, three profiles were extracted from the burnout scores during COVID-19.

Table S4. Comparisons between the models identified using the Latent Profile Analysis performed to assess the number of burnout profiles during COVID-19 to be extracted

Model	Profile	n	%	Entropy	Bootstrapped likelihood ratio test		
					Log-likelihood	Log-likelihood difference*2	p-value
2 profiles	1	78	20.6	.84	-3532.59	-	-
	2	299	79.4				
3 profiles	1	148	39.3	.80	-3438.15	74.58	<0.001
	2	176	46.6				
	3	53	14.1				
4 profiles	1	139	36.9	.81	-3400.85	27.85	<0.001
	2	55	14.7				
	3	11	2.90				
	4	172	45.6				

The analysis of the longitudinal measurement invariance showed that constraining the means of the MBI subscales to be equal over time worsened the model fit (chi-square difference test value = 362.79, df = 9, p<0.001). The absence of measurement invariance was deemed as theoretically plausible since the advent of the pandemic might have influenced the characteristics of the burnout profiles. Therefore, the model without longitudinal measurement invariance was retained.

Role of HSE perceived work stressors and work satisfaction on transition probabilities from Engaged or Ineffective to Overextended or Severe Burnout

Table S5 displays the Odd Ratios (OR) with 95% Confidence Intervals from the multinomial logistic regression model performed to assess the associations between work stressors and the transitions from the Engaged or Ineffective profiles before COVID-19 to the Overextended or Severe burnout during COVID-19.

Table S5. Relationships between HSE perceived work stressors and work satisfaction with the transitions from the Engaged and Ineffective burnout profile before COVID-19 to the Overextended and Severe burnout profiles during COVID-19

		**Transition to Exhausted during COVID-19**		**Transition to Severe burnout during COVID-19[a]**	
		**OR**	**95%CI**	**OR**	**95%CI**
Demands	Engaged	1.03	0.96-1.11	0.63	0.34-1.18
	Ineffective	1.04	0.86-1.27	0.75	0.48-1.18
Control	Engaged	0.96	0.88-1.05	2.13	0.75-6.07
	Ineffective	0.96	0.75-1.24	1.04	0.72-1.48
Role clarity	Engaged	1.00	0.87-1.15	0.85	0.38-1.87
	Ineffective	1.00	0.69-1.45	0.51	0.24-1.09
Managers' support	Engaged	1.02	0.90-1.16	0.67	0.34-1.31
	Ineffective	1.67	1.08-2.59[b]	1.33	0.79-2.24
Peer support	Engaged	1.20	1.04-1.39[b]	0.52	0.16-1.65
	Ineffective	0.85	0.53-1.37	1.07	0.50-2.32
Hostile relationships	Engaged	1.17	1.03-1.32[b]	0.68	0.24-1.97
	Ineffective	1.45	0.90-2.35	1.62	0.75-3.49
Participation in work organization	Engaged	0.93	0.83-1.04	0.81	0.37-1.79
	Ineffective	0.97	0.69-1.38	0.66	0.41-1.04
Work satisfaction	Engaged	0.88	0.72-1.06	0.04	0.01-1.76
	Ineffective	0.37	0.17-0.81[b]	0.57	0.22-1.48

Note. The model included as predictors work stressors, work satisfaction, profile membership before COVID-19 and the interactions between work stressors and work satisfaction with profile membership before COVID-19.

[a] n=9 nurses or nurse assistants transitioned from Overextended to Severe burnout and n=3 from Engaged to Severe burnout

[b] = $p<.05$
